# *Anaplasmataceae*: Dichotomous Autophagic Interplay for Infection

**DOI:** 10.3389/fimmu.2021.642771

**Published:** 2021-04-12

**Authors:** LaNisha L. Patterson, Caitlan D. Byerly, Jere W. McBride

**Affiliations:** ^1^ Department of Pathology, University of Texas Medical Branch, Galveston, TX, United States; ^2^ Department of Microbiology and Immunology, University of Texas Medical Branch, Galveston, TX, United States; ^3^ Center for Biodefense and Emerging Infectious Diseases, University of Texas Medical Branch, Galveston, TX, United States; ^4^ Sealy Institute for Vaccine Sciences, University of Texas Medical Branch, Galveston, TX, United States; ^5^ Institute for Human Infections and Immunity, University of Texas Medical Branch, Galveston, TX, United States

**Keywords:** autophagy, xenophagy, *Anaplasmataceae*, *Ehrlichia*, *Anaplasma*, effector, autolysosome, phagolysosome

## Abstract

Autophagy is a vital conserved degradative process that maintains cellular homeostasis by recycling or eliminating dysfunctional cellular organelles and proteins. More recently, autophagy has become a well-recognized host defense mechanism against intracellular pathogens through a process known as xenophagy. On the host-microbe battlefield many intracellular bacterial pathogens have developed the ability to subvert xenophagy to establish infection. Obligately intracellular bacterial pathogens of the *Anaplasmataceae* family, including *Ehrlichia chaffeensis*, *Anaplasma phaogocytophilium* and *Orientia tsutsugamushi* have developed a dichotomous strategy to exploit the host autophagic pathway to obtain nutrients while escaping lysosomal destruction for intracellular survival within the host cell. In this review, the recent findings regarding how these master manipulators engage and inhibit autophagy for infection are explored. Future investigation to understand mechanisms used by *Anaplasmataceae* to exploit autophagy may advance novel antimicrobial therapies and provide new insights into how intracellular microbes exploit autophagy to survive.

## Introduction

Autophagy is a well characterized host defense mechanism in which invading microbes are tagged for degradation in a selective autophagic process known as xenophagy (1–7). Although xenophagy is a known host defense mechanism against invading microbes, various intracellular pathogens including obligately intracellular rickettsial pathogens in the family *Anaplasmataceae* can induce autophagy as a survival mechanism (2, 5–8). In contrast, evasion of the autophagic pathway is also a strategy utilized by intracellular pathogens for infection. Accumulating evidence provides insight regarding the dichotomous interplay that occurs between obligately intracellular bacteria and the autophagic pathway to promote infection.

In eukaryotic cells, autophagy is a highly conserved catabolic, lysosomal-dependent process that delivers long-lived proteins and damaged cytoplasmic components to the lysosome (9–12). At basal levels, autophagy plays an important role as a response to cellular stress and maintaining homeostasis through quality control of essential cellular components. Cellular homeostasis is maintained by degrading excessive, damaged, and/or aged proteins, peptides and organelles. Macroautophagy, the best described autophagy subtype, works to sequester damaged cytoplasmic components in a double-membrane vesicle known as the autophagosome (13). Macroautophagy can be further categorized into nonselective autophagy which randomly engulfs cellular components within the cytoplasm into autophagosomes for degradation upon fusion with a lysosome, and selective autophagy which degrades a specific type of cargo tagged for degradation (14). Below, we will summarize the major steps of the autophagic process and the major autophagy protein groups that regulate each step of the autophagic process. Furthermore, we will discuss critical findings linking these proteins with *Anaplasmataceae*-induced autophagy.

The autophagic process can be divided into distinct stages, including autophagy induction, phagophore formation and elongation, cargo recognition, autophagosome maturation, lysosomal fusion and autophagosome degradation (13, 15). In coordination with these steps are several major signaling pathways and autophagy-related genes (ATGs). mTOR kinase is a major player in the regulation of the autophagic process (16, 17). Wnt and phosphoinositide 3-kinase (PI3K)/ATP dependent tyrosine kinase (Akt) signaling pathways regulate mTOR (18, 19). The Wnt pathway plays an essential role in inhibition of autophagy by regulating activation of the mTOR pathway. mTOR activation occurs downstream of PI3k/Akt signaling to inhibit autophagy (20, 21). Additionally, glycogen synthase kinase-3 (GSK3) inhibits the mTOR pathway by phosphorylating tuberous sclerosis complex 2 (TSC2) in a manner dependent on AMPK phosphorylation (22). Importantly, TSC2 is a Rheb GTPase-activating protein, a Ras family GTPase and an mTOR activator (23).

mTORC1 inhibition leads to autophagy induction due to activation of AMPK signaling (24). Upon decreased mTORC1 activity, the initiation of phagophore formation is stimulated by activation of the class III phosphatidylinositol 3-kinase (PtdIns3K) complex. The Ulk1 protein complex signals the formation of the PtdIns3K complex, which includes, Beclin-1 (Atg6/Vps30), Vps34 (vacuolar protein sorting 34), Vps15 (p150, a myristoylated serine/threonine kinase), Ambra-1 (Autophagy/Beclin-1 Regulator 1), and ATG14 (13, 25, 26). Together, the ULK1 protein complex and the PtdIns3K complex integrate nutrient status (ULK1) with autophagosome formation (PtdIns3K) (25).

Beclin-1, an orthologue of the Atg6/vacuolar protein sorting Vps30 protein in yeast, plays a central role in autophagy. Beclin-1 is important for localization of autophagic proteins to the PAS to regulate the lipid kinase Vps34 protein and promote formation of Beclin-1/Vps34/Vps15 core complexes (13, 26). The formation of the Beclin-1/Vps34/Vps15 complex marks the initiation of autophagy (27). The PtdIns3K complex, along with other Atg proteins, also recruits two ubiquitin-like conjugation systems, Atg12/Atg5/Atg16 and Atg8-phosphatidylethanolamine (PE), to the phagophore to recruit Atg8-PE machinery and regulate membrane elongation and expansion of the autophagosome (26, 28, 29).

Atg5/Atg12/Atg16 conjugation complex has been shown to lead to conjugation of microtubule-associated protein 1 light chain 3 (LC3), to the membrane of the autophagosome (29). This leads to the conjugation of LC3-I to phosphatidylethanolamine (PE) to form LC3-II. The p62/SQSTM1 (sequestosome 1) protein acts as a cargo receptor for ubiquitinated targets which are transported to the autophagosome for degradation (30, 31). Following phagophore expansion, the phagophore is completely sealed, forming the double membrane autophagosome containing all targeted components. Maturation of the autophagosome involves fusion with both early and late endosomes, which requires GTP bound small G protein Rab5, Rab7, and presenilin protein (32). The autophagosome fuses with the lysosome to form an autolysosome for degradation of engulfed components.

LC3-II and p62/SQSTM1 are also utilized as markers for autophagosome formation due to its degradation within the lysosome along with damaged and recycled components (33). Products, along with some of the autophagy cargo, are degraded by lysosomal hydrolases and recycled as amino acids supplements within the tricarboxylic acid cycle (TCA) cycle or as fatty acids, sugars, and proteins to increase energy for cell survival (9, 13).

### Autophagy as an Intracellular Innate Defense Pathway

Although studies have demonstrated autophagy as a host defense mechanism against bacterial pathogens, many intracellular pathogens have evolved strategies to subvert autophagy for survival (34–36). Autophagy is considered a downstream effector mechanism that plays an integral role in both innate and adaptive immunity to various pathogens (9, 37). Xenophagy is a selective autophagy whereby intracellular pathogens are tagged by ubiquitin and subsequent targeting to autophagosomes for degradation in autolysosomes. Autophagy receptors such as p62/SQSTM1, nuclear domain 10 protein 52 (NDP52) and neighbor of BRCA1 gene 1 (NBR1) have been shown to bind ubiquitinated intracellular pathogens for autolysosome destruction and clearance (31). Autophagy plays an important role in both innate and adaptive immunity to various intracellular pathogens including *Mycobacterium tuberculosis*, *Streptococcus pyogenes*, *Listeria monocytogenes*, and *Salmonella enterica* (38–42).

This review presents the current knowledge regarding the dichotomous interplay between rickettsial pathogens in the family *Anaplasmataceae*, namely *Ehrlichia chaffeensis*, *Anaplasma phaogocytophilium* and *Orientia tsutsugamushi, and* the autophagic pathway during infection. These rickettsial pathogens utilize secreted effector proteins and host signaling pathways to hijack the autophagic pathway for survival.

## *Anaplasmataceae*: Intracellular Pathogens of Life-Threatening Human Infections

Members of *Anaplasmataceae* are α-proteobacteria in the order of Rickettsiales that include genera *Anaplasma, Ehrlichia, Neorickettsia*, and *Orientia* (43–46). *Anaplasmataceae* family includes obligately intracellular bacteria that reside in membrane bound cytoplasmic vacuoles mainly within phagocytic cells and are transmitted primarily by arthropod vectors that acquire the infection from persistently infected vertebrate hosts. These pathogens are master manipulators of the host cells (arthropod and mammalian) in which they infect. Successful intracellular infection occurs by hijacking conserved cellular signaling pathways, reprogramming host cell gene transcription, and by exploitation of other cellular processes to subvert host defense mechanisms including autophagy.

*Anaplasmataceae* members are best recognized for causing tick borne emerging life-threatening zoonotic diseases in the United States. Human monocytotropic ehrlichiosis (HME) and human granulocytic anaplasmosis (HGA) are group I NIAID tick-borne zoonoses caused by *E. chaffeensis* and *A. phagocytophilum*, respectively (47, 48)*. E. chaffeensis* is maintained in nature by persistent infection of white-tailed deer, which is the primary mammalian reservoir. *E. chaffeensis* is transmitted by the lone star tick, *Amblyomma americanum*, which maintains the infection transstadially (1–3). *Anaplasma phagocytophilum* transstadially infects *Ixodes scapularis* ticks and other *Ixodes* spp. after acquiring the infection from infected small mammal reservoirs such as the white-footed mouse. In contrast, *O. tsutsugamushi* the etiologic agent of scrub typhus, a disease endemic to the Asian continent and present throughout Indonesia and northern Australia, is transmitted mainly by the bite of larva life stage-infected *Leptotrombidium* mites (49, 50). HME, HGA and scrub typhus have similar clinical presentations characterized by initial symptoms including fever, headache, myalgia, nausea, confusion, conjunctival injection (red eyes), and chills within the first two weeks following infection (1, 3, 15). Common laboratory abnormalities include thrombocytopenia, leukopenia, anemia, and elevated hepatic transaminases (2, 14, 16–18). Disease severity ranges from mild to life-threatening complications such as toxic shock-like syndrome, kidney failure, meningoencephalitis, and acute respiratory distress (1, 2, 4).

Members of the *Anaplasmataceae* family have small genomes but have evolved complex molecular strategies that enable them to create a permissive intracellular niche within professional phagocytes and other cells. Due to the obligately intracellular existence, *Anaplasmataceae* genomes have been shaped by a process known as reductive evolution resulting in loss of metabolic pathway genes that are no longer required for intracellular survival (51, 52). They replicate in membrane-bound cytoplasmic vacuoles within the host cell cytoplasm and undergo different developmental phases during infection. There are two well-defined ultrastructural forms, the dense-cored cell and reticulate cell, which have been identified by electron microscopy (53–56). The infectious dense-cored non replicating cell is small (0.4-0.6 µm), more electron dense, and has tightly coiled nucleoid DNA. In contrast, the reticulate cell is the replicative form and is larger (0.4-1.9 µm) with a dispersed nucleoid DNA. Dense-cored organisms interact with host cell receptors and enter the host cell by receptor-mediated endocytosis. After entry, dense-cored ehrlichiae transition into intermediate then full reticulate cell forms that replicate by binary fission, forming microcolonies known as morulae within host derived membrane-bound vacuoles. The ehrlichial replication cycle takes approximately 48 h, then the replicating reticulate cells transition into infectious dense-cored ehrlichiae which are released from the host cell by cell lysis or exocytosis to infect other cells (57, 58).

### Secretion Systems and Effectors

As with some Gram-negative bacteria, *Anaplasmataceae* have well known secretion systems that secrete effector proteins into the host cell. Type I, II and IV secretion systems have been identified in *Anaplasmataceae*. Notably, the type III secretion system found in some obligately intracellular bacteria (i.e., chlamydiae), is absent (59–61). These macromolecular secretion nanomachines are distinctly different in secretion mechanisms and the secreted effectors. Several bacterial effectors are known to regulate selective autophagy through various mechanisms for survival (62–69). Below are listed some of the secreted effector proteins by members of *Anaplasmataceae* that play a significant role in the subversion of autophagy.

The T1SS is well characterized in many extracellular bacteria and is known to secrete a number repeat-containing pore-forming toxins known as the Repeats in Toxin Family (RTX) (70, 71). The T1SS is widespread in Gram-negative bacteria and transports substrates in a one-step process across two membranes without any periplasmic intermediate into the extracellular space (72–75). Several T1SS substrates have been identified that are secreted by members of *Anaplasmataceae*, including ankyrin repeat (AR) and tandem repeat effector proteins. There are currently four characterized T1SS tandem repeat protein (TRP) effectors that have been identified in *E. chaffeensis*-infected cells including TRP32, TRP47, TRP75 and TRP120 (60, 76–78). TRPs are nucleomodulins and TRP120 has also been shown to activate host cell signaling pathways (Notch and Wnt) to downregulate innate defense mechanisms (79–82). Several of the TRPs have been shown to play a role in inhibiting TFEB nuclear localization and autolysosome generation during *E. chaffeensis* infection by reprogramming signal transduction pathways, including the Wnt signaling pathway (21). *E. chaffeensis* Ank200 is a nucleomodulin secreted by the T1SS that binds adenine-rich *Alu* elements in host promoter and intron regions (83, 84). *O. tsutsugamushi* secrete T1SS AR family members that traffic to diverse subcellular localizations including the endoplasmic reticulum (61). The *O. tsutsugamushi* (Ikeda strain) genome encodes 38 Ank-containing ORFs, each of which display characteristics consistent with T1SS effectors (LDAVTSIF residues found in 37-63% in their final 60 amino acids, acidic pI values and very few cysteines) (85). *O. tsutsugamushi* Anks modulate NF-κβ to enhance infection; however, there is no evidence that *O. tsutsugamushi* T1SS substrates manipulate the autophagic pathway (86). Notably, *A. phagocytophilum* T1SS effectors have not been identified to date.

The T4SS is a well characterized ATP-dependent, double membrane-spanning multiprotein secretion nanomachine found in both Gram-negative and -positive bacteria (87–90). The archetypal Gram-negative T4SS is defined by the plant pathogen Agrobacterium tumefaciens VirB/D4 system (91). In Rickettsiales, a homologous but structurally different T4SS system is present with *vir* genes organized in three genome locations (92). To date, there have been a total of six T4SS effector proteins identified between *E. chaffeensis* and *A. phagocytophilum;* however, functions for only five of these effectors have been reported (67, 93–97). *Anaplasma* translocated substrate 1 (Ats-1) and ankyrin repeat domain-containing protein A (AnkA) have been functionally characterized. Ats-1 is an orthologue of the *E. chaffeensis* T4SS effector protein, Etf-1. Both Ats-1 and Etf-1 play roles in subverting apoptosis and host autophagy for intracellular survival (66, 67, 94, 98). Etf-2 delays endosomal maturation to avoid routing *E. chaffeensis* to phagolysosomes (95). Another *E. chaffeensis* effector, ECH0825, is highly upregulated during early stages of infection during exponential growth in THP-1 human monocytic leukemia cells and has been shown to translocate to mitochondria where it inhibits reactive oxygen species production and host cell apoptosis by upregulating MnSOD, an essential mitochondrial antioxidant enzyme (93).

## Pathogen-Host Interactions

### *E. chaffeensis*

Utilizing TRP and other effectors, *E. chaffeensis* avoids host immune defenses of the mononuclear phagocyte making it a remarkable model organism for examining novel host-pathogen interactions involved in cellular reprogramming. *E. chaffeensis* TRP effectors are secreted by the T1SS and translocate the vacuole membrane by an unknown mechanism to access the host cell. During infection, TRPs interact with a multitude of host proteins and elicit strong protective antibody responses to molecularly defined linear epitopes (99–102). TRPs are nucleomodulins that translocate to the host cell nucleus through a noncanonical NLS-independent mechanism (79–81). In addition, TRP120 has other defined functional roles during infection, and thus, is considered a moonlighting protein. These roles include promoting ehrlichial entry (103, 104), activation of host signaling pathways through ligand mimicry (99, 105), nucleomodulin activity (106–108), and as a HECT E3 ubiquitin ligase that targets host substrates for degradation (106, 107, 109, 110). The ability of *E. chaffeensis* to interface with the host cell is known to involve post-translation modifications including sumolyation (104), ubiqiuitination (109) and others (104). *E. chaffeensis* appears to exploit host cell machinery to acquire post translational modifications (PTMs) in some instances, but ehrlichial encoded ubiquitin ligases such as TRP120 are involved in creating PTMs that play a role in host-pathogen interplay (109). *E. chaffeensis* gene knockout studies have shown that TRP120 is essential for *E. chaffeensis* survival *in vivo* which can be attributed to the many defined functions of TRP120 and highlights the major role TRP120 plays in infection and survival (58).

### *A. phagocytophilum*

*A. phagocytophilum* utilizes an array of bacterial proteins for adherence, invasion, and survival within the host cell. Infection is known to depend on numerous type IV secreted effector proteins, transmembrane proteins, surface proteins, and *A. phagocytophilum*-occupied vacuole membrane (AVM) proteins. These proteins include major surface protein 4 (MSP4), nucleomodulin AnkA, adhesin protein Asp14, and heat shock protein 70 (HSP70). The nucleomodulin AnkA binds host DNA and protein complexes within the nucleus of neutrophils to alter gene transcription (111). Ats-1 plays a role in preventing apoptosis by stabilizing mitochondria through the disruption of Bax-induced apoptosis to promote *A. phagocytophilum* infection (94).

PTMs are also involved in pathogen*-*host interactions. Effector protein APH0032 decorates the AVM interface and is a sumoylated by co-opting host SUMO machinery during infection. Similarly, *A. phagocytophilum* protein A (AmpA) is a critical effector protein that is also sumoylated to promote infection. AmpA localizes to the AVM throughout infection colocalizing with SUMO 2/3 and SUMO1 as the infection progresses (112). The nucleomodulin AnkA binds host DNA and protein complexes within the nucleus of neutrophils to alter gene transcription (111).

### *O. tsutsugamushi*

*O.* *tsutsugamushi* encodes multiple T1SS ankyrin-repeat-containing effector proteins (Anks), known to interact with host cells and largely target the endoplasmic reticulum (113). Notably, Ank9 was the first effector shown to function during infection whereby a unique Ank9 motif mimics the GRIP domain of the host golgins, supporting *O.* *tsutsugamushi* localization to the host Golgi. Ank9 binds host protein COPB2 to hijack the endoplasmic reticulum *via* retrograde trafficking from the Golgi. Following its translocation, Ank9 activates the transcription factor 4-dependent unfolded protein response to support *O. tsutsugamushi* infection (114). Studies have revealed *O. tsutsugamushi* nucleomodulins Ank1 and Ank6 abrogate NF-kB-activated transcription utilizing exportin-1 independent mechanisms to decrease TNFα-induced p65 nuclear levels (86).

## *Anaplasmataceae*-Mediated Exploitation of Conserved Host Cell Signaling Pathways

Conserved signaling pathways Wnt and Notch which play an important role in regulating innate host defenses, including phagocytosis, autophagy, and toll-like receptor (TLR) expression are exploited by *E. chaffeensis* for intracellular survival (20, 115–117). The Wnt/β-catenin signaling pathway regulates both basal and stress-induced autophagy (20). β-catenin suppresses autophagosome formation and directly suppresses p62/SQSTM1 through T-cell factor 4 (TCF4), one of the transcriptional factors in the Wnt signaling pathway (20). Autophagy has also been shown to inhibit Notch signaling though modulation of the PTEN-PI3K/Akt/mTOR pathway (115). Inhibition of Wnt and Notch signaling dramatically reduces *E. chaffeensis* infection demonstrating the importance of the conserved cell signaling pathways for persistent infection and survival (105, 118). Notably, *E. chaffeensis* hijacks the canonical and non-canonical Wnt signaling pathways *via* effector proteins to promote infection (58). In addition, nucleomodulins TRP32, TRP47, and TRP120 bind DNA motifs within the promoter regions of Wnt target genes and may modulate Wnt gene transcription (118). Furthermore, yeast-two-hybrid (Y2H) analysis has identified protein-protein interactions between *E. chaffeensis* effector proteins (TRP32 and TRP120) and host proteins involved in Wnt signaling and transcriptional regulation of Wnt genes (99, 101). Of those identified, were interactions with Wnt signaling negative regulators (CEP164, KLHL12, ILF3 and LMO2) and positive regulators (PPP3R1 and VPS29) (99, 101).

TRP120 interaction with the host cell facilitates entry and this appears to occur *via* activation of non-canonical Wnt signaling resulting in Ca^2+^ signaling and triggering uptake through phagocytosis (118). Wnt signaling has been shown to enhance infection as RNA silencing of Wnt signaling components, including β-catenin, NFAT, CK1, and CAMKII significantly reduces *E. chaffeensis* infection, indicating that Wnt signaling is required to maintain infection (118). RNA silencing of the ubiquitously expressed Fzd5 and Fzd9 Wnt receptors, as well as the Wnt co-receptor LRP6 also results in reduced infection, indicating a possible role of the receptors for *E. chaffeensis* entry into the host. More specifically, RNA silencing of Fzd5 or its ligand Wnt5a results in a highly significant reduction of infection, suggesting the necessity of Wnt5a-Fzd5 signaling for *E. chaffeensis* entry and survival.

The Notch signaling pathway is an evolutionarily conserved pathway with critical roles in cellular homeostasis, cell proliferation and differentiation; however, Notch activation has also been shown to have significant roles in MHC class II expansion, B and T cell development, and regulation of innate immune mechanisms such as autophagy and apoptosis (119). Recently, Notch activation by *E. chaffeensis* was shown to downregulate TLR2/4 expression (105). Interestingly, TRP120 was identified as a Notch ligand mimic resulting in Notch activation as shown by nuclear translocation of the Notch intracellular domain (NICD), a hallmark for Notch activation. TRP120 is also a HECT E3 ubiquitin ligase that ubiquitinates Notch negative regulator FBW7 for proteasomal degradation resulting in increased oncoproteins levels including induced myeloid leukemia cell differentiation protein (MCL1) and NICD (107). Collectively, the data demonstrate that exploitation of conserved signaling pathways, such as Wnt and Notch is a major strategy involved in ehrlichial survival and possibly other members of the *Anaplasmataceae* family by modulating autophagy and other innate host defense mechanism.

## *Anaplasmataceae* Effector-Induced Autophagy for Nutrient Acquisition

**Figure 1:**
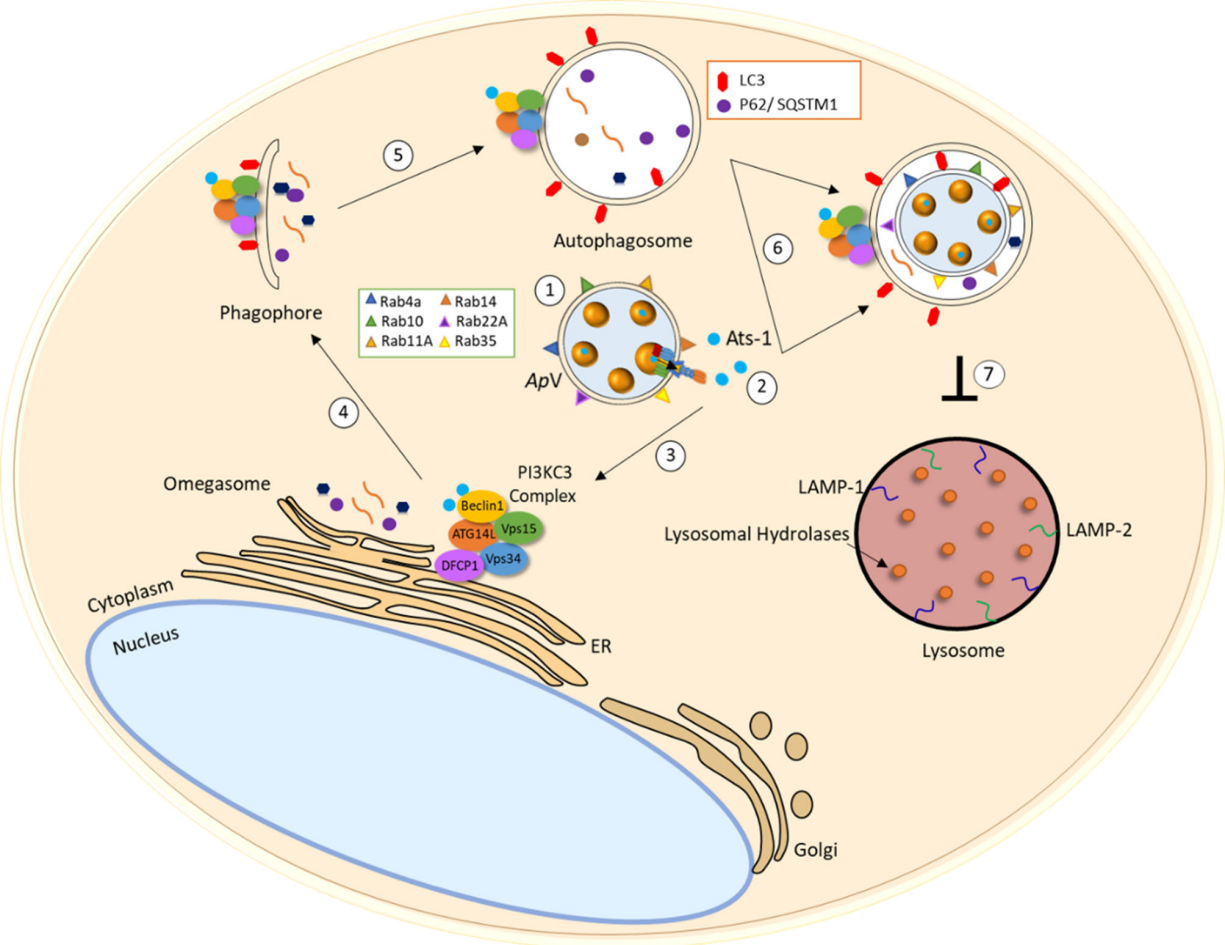
*A. phagocytophilum* interplay with the autophagic pathway. (1) *A. phagocytophilum* selectively recruits Rab GTPases Rab4A, Rab10, Rab11A, Rab14, Rab22A, Rab35 which regulate endocytic recycling and Rab1 which regulates vesicular protein transport from the endoplasmic reticulum (ER) to the Golgi compartment. (2) T4SS effector Ats-1 is translocated from the ApV into the host cell cytoplasm and (3) directly interacts with autophagsome initiation complex (Atg14-Beclin 1-Vps34) to initiate omegasome formation in the ER. (4) Isolation membrane elongates and (5) double-membrane autophagosome decorated with LC3 form. (6) Autophagosomes are recruited to the ApV that fuse to release autophagic body content. (7) *A. phagocytopilum* blocks lysosomal fusion potentially by preventing endosomal maturation and/or through other unknown mechanisms.

**Figure 2:**
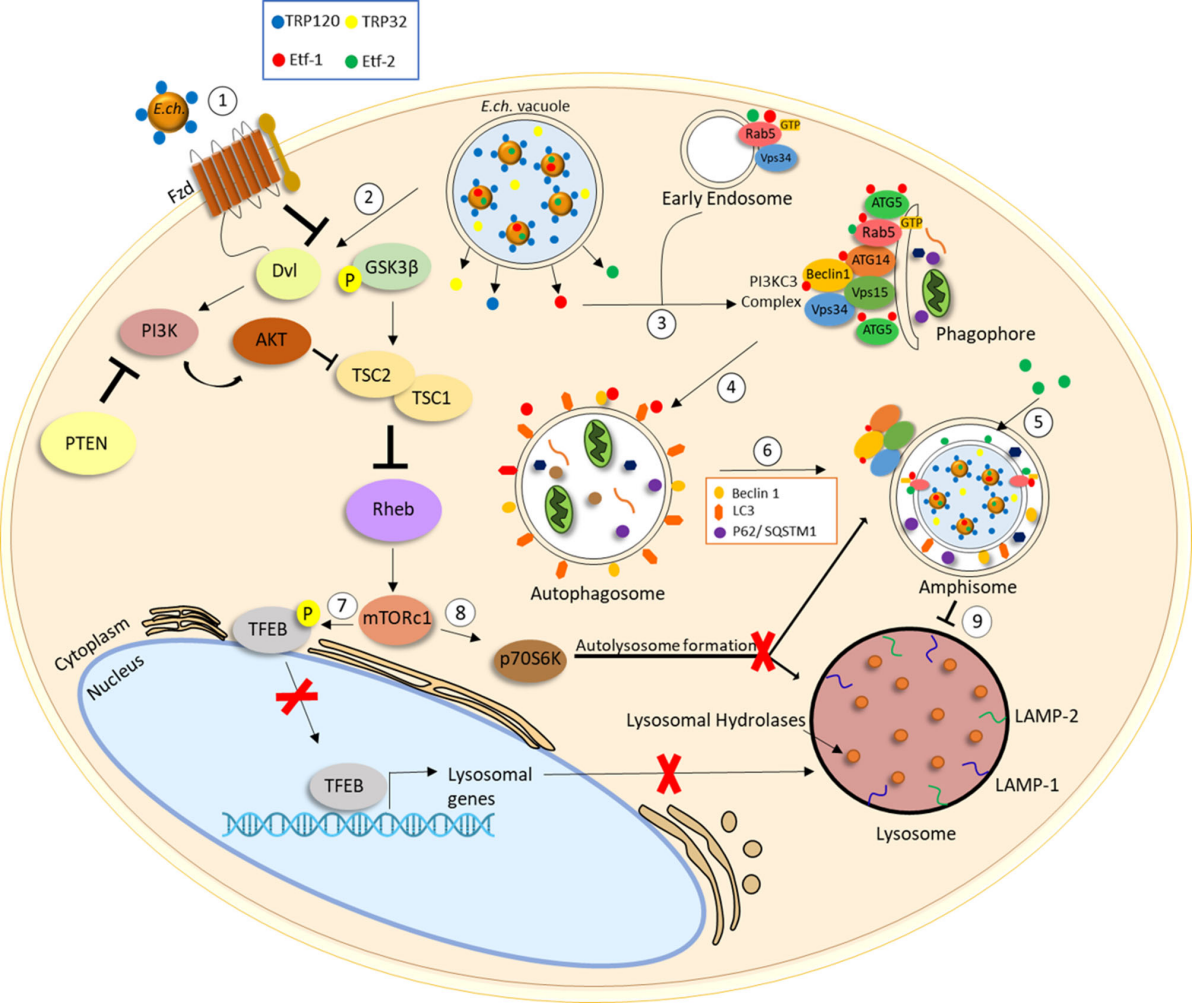
*E. chaffeensis* interplay with the autophagic pathway. (1) *E. chaffeensis* dense-cored cells express effectors important for Wnt signaling including T1SS effectors TRP120 and TRP32. *E. chaffeensis* stimulates phagocytosis for entry through interaction between TRP120 and the Fzd receptor/co-receptor complex. (2) *E. chaffeensis-*mediated Wnt-PI3K/Akt signaling stimulates increased levels of phospho-GSK3-β reducing TSC2 and increasing Rheb activity leading to mTOR activation. (3) *E. chaffeensis* T4SS effector Etf-1 is secreted into the host cell cytoplasm and interacts with Beclin1, PI3CK complex and Rab5-GTP to stimulate phagophore formation. (4) ATG5 and LC3 engage to induce autophagosome formation in a class III PtdIns3K-dependent manner. (5) *E. chaffeensis* T4SS effector Etf-2 localizes to *E. chaffeensis* vacuole membrane and binds to RAB5-GTP to delay endosome maturation. (6) Autophagosomes displaying Beclin1, LC3II and p62/SQSTM1 fuse with *E. chaffeensis* inclusions to form amphisomes. (7) mTOR activation leads to TFEB phosphorylation and inhibition of TFEB nuclear translocation. Inhibition of TFEB nuclear translocation prevents transcription of genes involved in lysosomal biogenesis and (8) increased phospho-p70 S6 kinase activity inhibits autolysosome formation.

*Anaplasmataceae* are auxotrophic with a limited capacity to synthesize required nutrients for survival, and thus, obtain essential nutrients from the host cell. Both *A. phagocytophilum* and *E. chaffeensis* survive by replicating within a host cell-derived membrane bound vacuole. Autophagosomes are induced by *E. chaffeensis* and *A. phagocytophilum* secreted T4SS effector proteins, Etf-1 and Ats-1, respectively (67, 94) (**Figures 1** and **2**). Etf-1 and Ats-1 are secreted into the host cell cytoplasm where they nucleate autophagosome formation. This effector-induced autophagosome formation is independent of mTOR activity. Ultimately, the effector generated autophagosomes fuse with the pathogen occupied vacuoles to deliver host-derived components. Ats-1 and Etf-1 induce autophagy in a class III PtdIns3K-dependent manner and localize to inclusions with autophagosomal markers. *A. phagocytophilum* inclusions colocalize with early autophagosomal markers Beclin 1 and Vps34-Atg14 and are enveloped by double‐lipid bilayer membranes (**Figure 1**) (94). Furthermore, Ats-1 directly binds to Beclin 1 and induces autophagosome formation in an ATG14 dependent manner (53); however, no interaction appears to occur with Ats-1 and UVRAG, an autophagy protein that regulates autophagosome maturation.

Studies have shown that *E. chaffeensis* autophagosome nucleation is dependent on Rab5-GTP and Rab5-regulated trafficking for the biogenesis of *E. chaffeensis* vacuoles (**Figure 2**) (89). Etf-1 is known to bind Rab5, Beclin 1 and phosphatidylinositol 3-kinase (PI3KC3) to induce Rab5-regulated autophagy. Furthermore, *E. chaffeensis* ATG5 and Etf-1 were shown to localize to the membrane of inclusions and are essential for infection (54). Importantly, Etf-1 activates class III PtdIns3K, localizes with ATG5 and LC3, and interacts with RAB5-GTP, PI3CK and Beclin 1 to form a multimeric complex that fuses with *E. chaffeensis* inclusions. Collectively, these findings show that Etf-1 facilitates induction of RAB5-GTP autophagy through PI3CK and Beclin 1 recruitment, as well as class III PtdIns3K and ATG5 localization to *E. chaffeensis* inclusions. Importantly, *Ehrlichia*-containing vacuoles contain the late endosomal marker RAB7, as shown by mass spectrometry and confocal microscopy, but do not fuse with lysosomes (**Figure 2**) (120, 121).

*O. tsutsugamshi* induces autophagy during infection but actively escapes from autophagic destruction in dendritic cells (122). *O. tsutsugamshi* significantly increased endogenous LC3-II protein levels in phagocytic and nonphagocytic cells during early infection, however no significant colocalization of the bacteria and LC3-postive autophagosomes occurs (122, 123). Autophagy induction does not affect growth of *O. tsutsugamshi* as demonstrated by 3MA or rapamycin (autophagy inducer) treatment or use of atg3-knockout mouse embryonic fibroblasts (Atg3^-/-^ MEFs) (123). Therefore, unlike *E. chaffeensis* and *A. phagocytophilum, O. tsutsugamshi* induces autophagy; however, evades autophagosomal degradation by actively escaping from host autophagosomes. Currently, the mechanism of evasion is unknown, but is predicted to be mediated by bacterial gene expression or bacterial effector proteins.

While some pathogens hijack the autophagic pathway to replicate intracellularly, pathogens of the *Anaplasmataceae* family exploit autophagy and specific ATG proteins to acquire nutrients. However, exactly how pathogens of the *Anaplasmataceae* family manipulate autophagy proteins for exploitation is still unknown. Ats-1 and Etf-1 are orthologous proteins that may subvert autophagy through a similar sequence to exploit specific autophagy proteins important for nutrient acquisition (124). Additionally, some bacteria escape host autophagy through inhibition of autophagy induction. For example, *S. Typhimurium* inhibits autophagy initiation through regulation of the AMPK-dependent activation pathway of mTOR, while *M. tuberculosis* inhibits autophagy induction by disruption of JNK-ROS (reactive oxygen species) signaling pathway to avoid destruction (34, 36). In comparison, both *A. phagocytophilum* and *E. chaffeensis*, induce autophagy independent of mTOR to acquire nutrients and remodel their vacuoles. This mechanism is regulated by T4SS effector proteins regulating key host autophagy proteins involved in the initiation step of autophagy.

## *Anaplasmataceae* Prevent Endosomal Maturation to Avoid Lysosomal Fusion

*E. chaffeensis* occupied vacuoles have features of early endosomes including RAB5, transferrin receptor (TFRC), early endosome antigen 1 (EEA1), annexins I, II, IV and VI, clathrin heavy chain and α-adaptin (67, 121, 125). *A. phagocytophilum* selectively recruits Rab GTPases to avoid endosomal maturation and subsequent destruction by lysosomes (**Figure 1**) (126). *A. phagocytophilum* selectively recruits Rab GTPases that are primarily associated with recycling endosomes, including Rab4a, Rab10, Rab11A, Rab14, Rab22A and Rab35. Rab1 which mediates endoplasmic reticulum to Golgi apparatus trafficking, is also recruited to the *A. phagocytophilum* vacuoles (ApV). Selectivity of Rab GTPases is shown to be dependent on *A. phagocytophilum* protein synthesis, allowing the ApV to disguise itself as a host recycling endosome. Importantly, the ApV does not mature along the endocytic pathway or resemble early endosomes due to the lack of endosomal markers including RAB5, transferrin receptor (TFRC), early endosome antigen 1 (EEA1), annexins I, II, IV and VI, clathrin heavy chain and α-adaptin (127). Additionally, *A. phagocytophilum* inclusions are not acidic and do not acquire the late endosomal markers, including myeloperoxidase, CD63, LAMP-1 and V-type H+ ATPase. Therefore, *A. phagocytophilum* hijacks Rab GTPases and host cell membrane traffic pathways to disguise the ApV as a recycling endosome to avoid endosomal maturation and subsequent lysosomal fusion.

Etf-2, another T4SS *E. chaffeensis* secreted protein effector, localizes to *E. chaffeensis* vacuoles, binds to RAB5-GTP and delays endosome maturation (**Figure 2**) (21). Etf-2 contains a Tre2-Bub2-Cdc16 (TBC) domain lacking Rab-GTPase activity, as well as an Arg and a Gln finger motif required for Etf-2 localization to the endosomal membrane, resulting in delayed maturation of phagosomes to phagolysosomes. EtpE is an *E. chaffeensis* outer membrane protein that functions as an invasion to mediate host cell entry. The C-terminal fragment of EtpE (EtpE-C) appears to be primarily responsible for *E. chaffeensis* binding and entry. The phagocytosis of EtpE-C-coated latex beads in Etf-2-GFP transfected cells was significantly reduced in comparison to GFP-transfected control cells. RAB5, but not RAB7, was shown to localize to a significant amount of EtpE-C-coated latex bead containing phagosomes for a prolonged period, and no late endosomes and phagolysosomes were detected in Etf-2-GFP transfected cells, indicating delayed endosomal maturation. Etf-2 also prevents RABGAP5 localization to endosomes (95). Therefore, Etf-2 participates in blocking endosomal maturation and fusion with lysosomes to promote ehrlichial infection. Other pathogens have been shown to selectively block maturation of autophagosomes through various mechanisms, including avoidance of RAB7 recruitment (69, 128–131). Importantly, studies have indicated that Rab7 is essential for autophagosome maturation in general (132). Selective modulation of RAB5 function by *E. chaffeensis* Etf-2 leading to alterations in the autophagosome explains the selectivity in autophagosome maturation. In comparison, how the ApV recruits and hijacks specific Rab-GTPases is still unknown. Identification of *A. phagocytophilum* effector proteins that interact with Rab-GTPases associated with the ApV is critical.

Preventing lysosomal fusion is a common strategy that underlies pathogen survival. *A. phagocytophilum and E. chaffeensis* vacuoles fuse with autophagosomes to form intermediate organelles. *E. chaffeensis* intermediate organelles have been described as amphisomes (**Figures 1** and **2**) (21, 98). Autophagosome markers Beclin 1 and LC3/GABARAP were found to colocalize with ehrlichial vacuoles indicating the fusion between autophagosomes and inclusions. Curiously, differences in LC3II localization to *E. chaffeensis* vacuoles have been reported. Rikihisa et al. reported no LC3II localization to ehrlichial vacuoles in RF/6A cells; however, others have detected ehrlichial vacuole localization with LC3/GABARAP in both THP-1 and RF/6A *E. chaffeensis*-infected cells (95). Moreover, significant increases in LC3II levels were observed during infection and consistent with those reported for *A. phagocytophilum*. Notably, colocalization of the *A. phagocytophilum* and *E. chaffeensis* inclusions with lysosomal markers, including LAMP-1 and LAMP-2 were not detected. Increased p62/SQSTM1 levels were also detected in *E. chaffeensis*-infected cells in comparison to control cells, as another indication of inhibited lysosomal fusion. Collectively, *Anaplasmataceae* pathogens induce autophagy for nutrient acquisition but inhibit lysosomal maturation through selective recruitment and avoidance of specific Rab GTPases.

## *Ehrlichia*-Exploitation of Wnt Signaling to Inhibit Autolysosome Generation and Autophagic Destruction

Wnt and PI3k/Akt pathways are important for ehrlichial survival, and regulation of autophagy by Wnt signaling has been documented. *E. chaffeensis* utilizes TRP effectors to exploit both the Wnt and PI3k/AKT pathways to activate mTOR signaling and regulate TFEB nuclear translocation to inhibit lysosomal biogenesis and autolysosomal fusion with the pathogen occupied vacuole. *E. chaffeensis* activates the PI3k/Akt pathway, a regulator of mTOR (**Figure 2**) (21). PI3K/Akt phosphorylates various proteins involved in regulation of cellular processes such as proliferation, apoptosis, and autophagy (133). Phosphorylated PI3K and Akt levels increase in *E. chaffeensis* infected cells, while phosphatase and tensin homolog (PTEN), a PI3K/Akt pathway inhibitor, levels decrease (21). The role of mTOR signaling in ehrlichial infection was also confirmed by siRNA knockdown of Rheb, a GTPase that activates mTOR. siRNA knockdown of both Rheb and phospho-p70 S6 kinase decreased *E. chaffeensis* infection (21). Thus, the mTOR activity is required for *E. chaffeensis* survival.

The Wnt signaling also regulates the PI3K/Akt pathway. GSK3-β is as a common protein and mediates crosstalk between PI3K/Akt and Wnt signaling pathways. More specifically, GSK3-β regulates mTOR by induction of Tuberous Sclerosis 2 Protein (TSC2) through phosphorylation and is also a negative regulator of Wnt/β-catenin (22, 134–136). Increased levels of GSK3-β were detected in -infected cells. These effects were abrogated with treatment of a Wnt-Dvl inhibitor (21). Additionally, inhibition of Akt and induction of GSK3 resulted in a significant decrease in infected cells at early and late infection intervals. Increased levels of phospho-GSK3-β were shown to be stimulated by T1SS effectors TRP120 and TRP32 (21). Therefore, TRP effectors activate the PI3K/Akt pathway and inhibit GSK3 activity by phosphorylation. Decreased levels of TSC2 were also shown in *E. chaffeensis*-infected cells. Collectively, these findings demonstrate activation of the PI3K/Akt pathway, phosphorylation and inactivation of GSK3 and inhibition of TSC2 during *E. chaffeensis* infection.

Phosphorylation and inactivation of GSK3, as well as inhibition of TSC2, results in activation of mTORC1 and subsequent phosphorylation and inhibition of TFEB nuclear translocation. TFEB is a transcription factor that coordinates expression of lysosomal hydrolases, membrane proteins and genes involved in autophagy signaling (**Figure 2**). TFEB was demonstrated to remain localized in the cytoplasm during *E. chaffeensis* infection and was confirmed to be mediated by *E. chaffeensis* Wnt activation. These finding support the conclusion that *E. chaffeensis* exploits Wnt-PI3K-/mTOR signaling in part to regulate mTOR signaling and TFEB nuclear localization to inhibit autolysosomal generation and promote ehrlichial survival.

Various studies have demonstrated the inhibition of autolysosome generation and autophagic destruction. *M. tuberculosis* inhibits Rab7 recruitment on Mtb-containing autophagosomes, while other pathogens neutralize lysosomal pH (69, 131, 137). This is the first study to elucidate the mechanism of *E. chaffeensis* inhibition of lysosomal fusion and, ultimately, destruction in the autolysosome. *E. chaffeensis* modulating conserved signal transduction pathways, including Wnt and Notch, to inhibit autolysosome generation may be applicable to other *Anaplasmataceae* bacterial pathogens.

## *Ehrlichia* Selective Autophagic Destruction Mediated by Antibody-TRIM21 Complex

**Figure 3:**
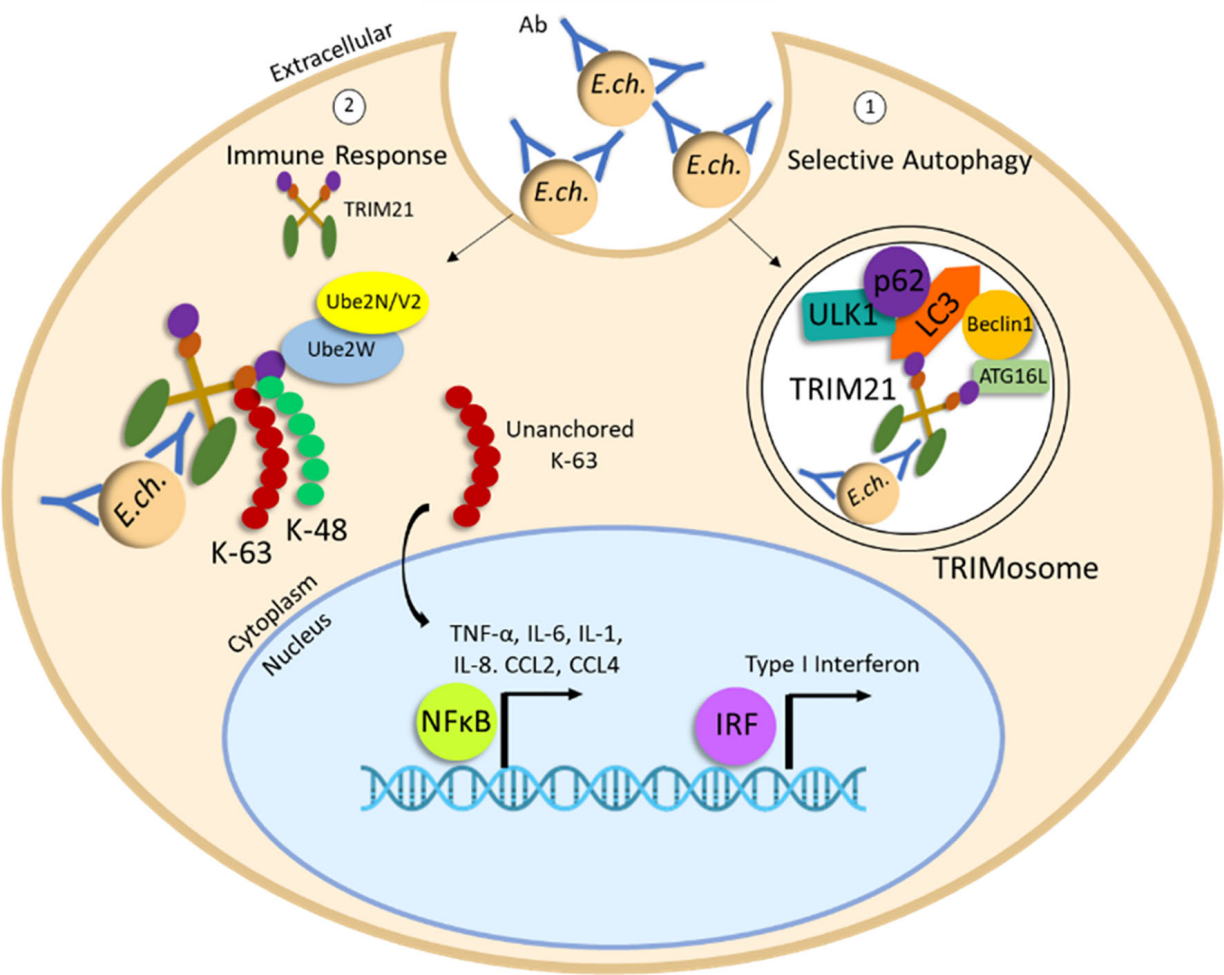
Degradation of *E. chaffeensis* by antibody-TRIM21-mediated selective autophagy. Ehrlichiae opsonized with *E. chaffeensis*-OMP-1 specific antibody are internalized by unknown uptake mechanism. Intracellular antibody-opsonized ehrlichiae are recognized by cytosolic Fc receptor TRIM21. (1) Induction of TRIMosome formation and selective autophagy occurs through recruitment of autophagy regulators, ULK1, Beclin 1, ATG16L and autophagy effectors LC3/GABARAP and p62/SQSTM1. (2) *E. chaffeensis*-Ab/TRIM21 complex stimulates rapid immune signaling and a proinflammatory response through accumulation of K48 and K63 polyUb chains and activation and nuclear translocation of NF-κB and IRF.

Antibody-mediated immunity to *E. chaffeensis* is well documented involving the classical antibody Fc receptor-dependent mechanism (**Figure 3**). However, intracellular antibody opsonized *E. chaffeensis* complexes engage TRIM21, an intracellular Fc receptor (138). Antibody opsonized ehrlichiae-TRIM21 complexes recruit autophagy regulators, ULK1, Beclin 1 and autophagy effectors LC3/GABARAP and p62/SQSTM1 resulting in proinflammatory responses and localized selective autophagic degradation of the ehrlichiae-antibody complexes. These findings demonstrate the importance of autophagy engagement of adaptive immune mechanisms and provide the first example of autophagic elimination of an intracellular pathogen by a TRIM21-mediated mechanism.

## Conclusions and Future Directions

Obligately intracellular pathogens of the *Anaplasmataceae* family have evolved highly sophisticated strategies to circumvent host immune response during infection. Autophagy is a cellular process targeted by microbial pathogens to promote infection. Mechanisms used by *Anaplasmataceae* for dichotomous engagement and subversion of autophagy for intracellular survival provides insight into the interplay that exists between the autophagic pathway and intracellular pathogens. Common amongst members of *Anaplasmataceae* are effector-mediated initiation of autophagy and the ability to hijack autophagy (ATG) proteins responsible for initiation and activation of the autophagic process. In contrast, effector interference with endosomal maturation contributes to pathogen survival. The ability to inhibit lysosomal destruction is a common theme demonstrated by *Anaplasmataceae*. This mechanism involves activation of cellular pathways such as Wnt and altering the pathogen vacuole to prevent lysosomal biogenesis and autolysosome generation. Studies to understand how *Anaplasmataceae* exploit the autophagic process may provide new insight; however, it is still unclear how intracellular pathogens out-compete the host for autophagy by-products and how autophagy by-products are obtained by *Anaplasmataceae* pathogens. It is also important to note that autophagy is an anti-inflammatory process, and it is therefore possible that Anaplasmataceae bacterial pathogens may strive to inhibit inflammation through activation of the autophagic pathway. Furthermore, very few details on the specific mechanisms that enable *A. phagocytophilum* and *O. tsutsugamshi* escape destruction by autophagy have been elucidated and need further investigation. Understanding mechanisms involved in autophagy induction and inhibition will inevitably help define how intracellular microbes exploit autophagy and could lead to novel antimicrobial therapeutic approaches.

## Data Availability Statement

The original contributions presented in the study are included in the article/supplementary material. Further inquiries can be directed to the corresponding author.

## Author Contributions

LP and JM conceptualized the work. LP gathered information and contributed all sections. CB contributed to sections pertaining to *O. tsutsugamushi* and exploitation of Wnt signaling. LP performed artwork. All authors contributed to the article and approved the submitted version.

## Funding

This work was supported by the National Institutes of Health grants AI149136, AI137779, and AI123610 awarded to JM, NIH 1F31AI152424-01 fellowship to LP, and T32AI007526-20 biodefense training fellowship to CB.

## Conflict of Interest

The authors declare that the research was conducted in the absence of any commercial or financial relationships that could be construed as a potential conflict of interest.
